# Generic and Respiratory-Specific Quality of Life in Non-Hospitalized Patients with COVID-19

**DOI:** 10.3390/jcm9123993

**Published:** 2020-12-09

**Authors:** Roy Meys, Jeannet M. Delbressine, Yvonne M.J. Goërtz, Anouk W. Vaes, Felipe V.C. Machado, Maarten Van Herck, Chris Burtin, Rein Posthuma, Bart Spaetgens, Frits M.E. Franssen, Yvonne Spies, Herman Vijlbrief, Alex J. van’t Hul, Daisy J.A. Janssen, Martijn A. Spruit, Sarah Houben-Wilke

**Affiliations:** 1 Department of Research and Development, Ciro, 6085 NM Horn, The Netherlands; Jeannetdelbressine@ciro-horn.nl (J.M.D.); yvonnegoertz@ciro-horn.nl (Y.M.J.G.); anoukvaes@ciro-horn.nl (A.W.V.); felipemachado@ciro-horn.nl (F.V.C.M.); maarten.vanherck@uhasselt.be (M.V.H.); reinposthuma@ciro-horn.nl (R.P.); fritsfranssen@ciro-horn.nl (F.M.E.F.); daisyjanssen@ciro-horn.nl (D.J.A.J.); martijnspruit@ciro-horn.nl (M.A.S.); Sarahwilke@ciro-horn.nl (S.H.-W.); 2 Nutrim School of Nutrition and Translational Research in Metabolism, 6229 HX Maastricht, The Netherlands; 3 Department of Respiratory Medicine, Maastricht University Medical Centre (MUMC+), 6229 HX Maastricht, The Netherlands; 4 REVAL–Rehabilitation Research Center, BIOMED–Biomedical Research Institute, Faculty of Rehabilitation Sciences, Hasselt University, 3500 Diepenbeek, Belgium; chris.burtin@uhasselt.be; 5 Department of Internal Medicine, Maastricht University Medical Centre (MUMC+), 6202 AZ Maastricht, The Netherlands; bartholomeus.spaetgens@mumc.nl; 6 Lung Foundation Netherlands, 3818 LE Amersfoort, The Netherlands; yvonnespies@longfonds.nl (Y.S.); hermanvijlbrief@longfonds.nl (H.V.); 7 Department of Pulmonary Diseases, Radboud Institute for Health Sciences, Radboud University Medical Center, 6525 GA Nijmegen, The Netherlands; Alex.vantHul@radboudumc.nl; 8 Department of Health Services Research, Care and Public Health Research Institute, Faculty of Health, Medicine and Life Sciences, Maastricht University, 6229 ER Maastricht, The Netherlands

**Keywords:** COVID-19, quality of life, Severe Acute Respiratory Syndrome Coronavirus 2 (SARS-CoV-2)

## Abstract

The impact of coronavirus disease 2019 (COVID-19) on quality of life appears to be highly underestimated, especially in patients who have not been admitted to the hospital. Therefore, our aim was to assess respiratory-specific quality of life in addition to generic quality of life in former patients with confirmed/suspected COVID-19 who have never been admitted to the hospital. Members of an online Belgian social support group for patients with confirmed/suspected COVID-19 with persistent complaints, completed an online survey. The five-level EQ-5D (EQ-5D-5L) and the Clinical COPD Questionnaire (CCQ) were used to assess generic and respiratory-specific quality of life, respectively. Data of 210 non-hospitalized patients (88% women, 45 ± 11 years, 79 ± 17 days after symptom onset) were included in the analyses. Mean EQ-5D index and visual analogue scale (EQ-VAS) score was 0.62 ± 0.19 and 50.71 ± 18.87, respectively, with 40% of the patients demonstrating an EQ-5D index that was below the fifth percentile of normative values, indicating poor generic quality of life. The mean CCQ score was 2.01 ± 0.98 points, while 123 respondents (59%) had a total score ≥1.9 points, indicating poor respiratory-specific quality of life. The correlation between EQ-5D index score/EQ-VAS score and CCQ total score was moderate (r = −0.524 and r = −0.374; both *p* < 0.001). In conclusion, both generic and respiratory-specific quality of life are affected in non-hospitalized patients with COVID-19, approximately three months after the onset of symptoms. The combined use of the EQ-5D and the CCQ could identify the broad impact of COVID-19 on quality of life.

## 1. Introduction

In the vast majority of patients with coronavirus disease 2019 (COVID-19), hospital admission is not required as illness is relatively mild [1,2]. Many of these patients, who have started to gather in online social support groups, experience multiple symptoms for weeks [3], with growing concern about the potential long-term consequences of COVID-19 [4]. Previously, it has been shown that the estimated total burden of pandemic influenza H1N1 was substantial when compared to other infectious diseases; both in hospitalized and non-hospitalized patients [5]. Nevertheless, the consequences of viral infections remain poorly understood and the impact of COVID-19 on quality of life in patients who have not been admitted to the hospital may be highly underestimated. To date, only a few studies, focused on hospitalized patients, demonstrated a negative impact of (suspected) COVID-19 symptoms on generic quality of life [6,7]. Quality of life can be assessed using generic (i.e., intended for general use, irrespective of the underlying disease) or disease-specific health status questionnaires (i.e., related to a specific condition or group). Even though both validated and standardized generic and respiratory-specific quality of life questionnaires exist, the question remains which instrument is more adequate in patients with a new disease such as COVID-19. As pulmonary function may be impaired [8,9] and dyspnea is one of the most prevalent symptoms in patients with COVID-19 [3], it seems reasonable to hypothesize that the impact on respiratory-specific quality of life, which not only focuses on the state of the airways but also on the patients’ functional needs, is very prominent in these patients. Therefore, our objective was to assess respiratory-specific quality of life in addition to generic quality of life in non-hospitalized patients with COVID-19. Furthermore, the association between both questionnaires as well as their discriminative ability were evaluated.

## 2. Materials and Methods

This is an analysis of a larger cross-sectional study (NTR NL8705) conducted in the Netherlands and Belgium (Flanders). In the current study, only Flemish patients were included in the analyses, due to the fact that these patients received an extended version of the survey including the assessment of respiratory-specific quality of life. Participants were recruited from an online Belgian social support group on Facebook [10] (~1200 members at time of assessment) for patients with confirmed/suspected COVID-19 with persistent complaints.

Participants were asked to fill in a web-based survey, which took approximately 30–35 min to complete. Patient representatives of the Dutch and Belgian Facebook groups as well as delegates of the ‘corona panel’ from the Lung Foundation Netherlands were closely involved in the development of the survey. Next to clinical characteristics and demographics (e.g., marital status, smoking status, level of education), questions regarding COVID-19 diagnosis and daily symptoms were included. Participants provided digital informed consent, were aged ≥18 years and understood the Flemish (Dutch) language. The Medical Ethics Committee of Hasselt University approved this study (CME2020/041).

### 2.1. Generic Quality of Life

The EQ-5D-5L [11] was used to assess generic quality of life. The EQ-5D-5L assesses health in five dimensions (Mobility, Self-care, Usual activities, Pain/Discomfort and Anxiety/Depression). Each dimension has five different levels of response (1: no problems, 2: slight problems, 3: moderate problems, 4: severe problems, and 5: extreme problems/unable to) [11]. Combining the dimension-specific levels across the five dimensions provides distinct health states (e.g., 21111, meaning slight problems in the mobility dimension and no problems in any of the other dimensions). Based on these 5-digit codes, an index score is provided (according to the preferences of the general population of a country/region) which ranges from −0.329 (worst quality of life) to 1 (best quality of life), with a score <0 representing a health state worse than death [12]. The reference values of Grochtdreis and colleagues [13] were used to calculate the proportion of patients with an EQ-5D index below the 5th percentile (1.64 × standard deviation; SD) of the mean age- and gender-based reference values. Additionally, participants had to rate their current overall health on a visual analogue scale (EQ-VAS) ranging from 0 (worst imaginable health) to 100 (best imaginable health).

### 2.2. Respiratory-Specific Quality of Life

The validated Dutch version of the Clinical COPD Questionnaire (CCQ) [14] was included to assess respiratory-specific quality of life. The CCQ is a 10-item assessment tool divided into three domains: symptoms (dyspnea, cough, and phlegm), mental state (feeling depressed and concerns about breathing), and functional state (limitations in different activities of daily life due to respiratory symptoms). All questions use a seven-point scale, ranging from 0 (never/not limited at all) to 6 points (all the time/totally limited or unable to). The main outcomes are the CCQ total score (sum of all items divided by 10) and mean scores of the three separate domains, ranging from 0 to 6 points, with a higher value indicating lower quality of life [14]. To classify high symptom burden, a CCQ total score of ≥1.9 has been used as cut-point (based on a population of patients with chronic obstructive pulmonary disease) [15].

### 2.3. Statistical Analysis

Mean (SD), median (interquartile range) or proportions were calculated, as appropriate. Continuous variables were tested for normality. Non-hospitalized patients were categorized into three groups: confirmed COVID-19 (based on reverse transcription polymerase chain reaction (RT-PCR) test and/or chest computed tomography (CT) showing pulmonary abnormalities consistent with COVID-19); symptom-based COVID-19 (medical diagnosis, solely based on symptoms); and suspected COVID-19 (no test/medical diagnosis). Differences between groups were analyzed with analysis of variance (ANOVA), Chi square or Kruskal–Wallis tests, as appropriate. Spearman’s rank correlations were calculated between EQ-5D and CCQ outcomes. Strength of correlation was classified as follows: very weak (0–0.19), weak (0.2–0.39), moderate (0.4–0.59), strong (0.6–0.79) and very strong (0.8–1) [16]. All analyses were performed with SPSS 25.0 (Armonk, NY, USA) and *p*-values of ≤0.05 were interpreted as statistically significant. Figures were created using Graphpad Prism 9.0 (San Diego, CA, USA).

## 3. Results

The survey was completed by 229 persons. Respondents who were admitted to the hospital (*n* = 15), reporting that the onset of symptoms was less than three weeks ago (i.e., acute phase; *n* = 3) and one respondent with missing data on gender were excluded from the analyses, resulting in 210 non-hospitalized patients for final analyses (88% women, 45 ± 11 years of age; Table 1). The majority were highly educated (64%), had no comorbidities before the COVID-19 infection (62%) and had never smoked (74%). Forty-nine patients (23%) had a confirmed COVID-19 diagnosis based on CT/RT-PCR testing, whereas 105 patients (50%) were medically diagnosed with COVID-19 based on symptoms (Table 1). The mean time since the onset of COVID-19-related symptoms was longer in the group of patients with suspected COVID-19 compared to the other two subgroups (Table 1).

### 3.1. Generic Quality of Life Scores

**Figure 1:**
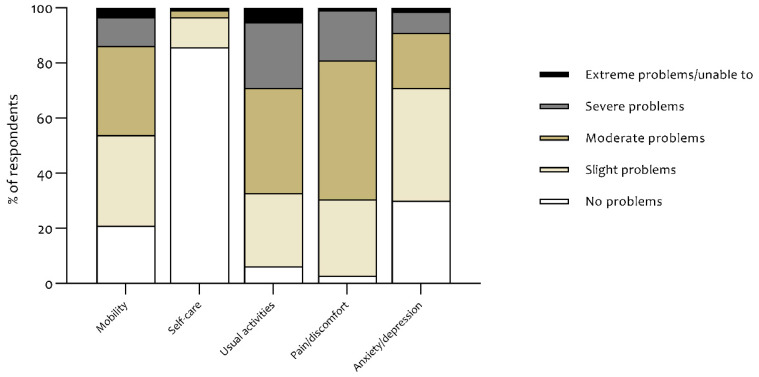
Distribution of responses to the descriptive system of the EQ-5D-5L, from which the EQ-5D index score is derived.

Mean EQ-5D index score was 0.615 ± 0.185 and ranged from 0.015 to 1.000. Compared to age- and gender-matched reference values [13], 40% of the patients had an EQ-5D index that was below the fifth percentile (Table 2). Mean EQ-VAS was 50.7 ± 18.9 (range: 0–99; Table 2). Figure 1 shows the distribution of responses to the EQ-5D-5L descriptive system. The usual activities and pain/discomfort dimensions showed the greatest self-reported impairment with 67% and 70% of the cohort reporting at least moderate problems, respectively. The self-care dimension was fairly unaffected, with 86% of the respondents reporting no problems. EQ-5D-5L frequencies and proportions reported by dimension and level are demonstrated in Table S1.

### 3.2. Respiratory-Specific Quality of Life Scores

**Figure 2:**
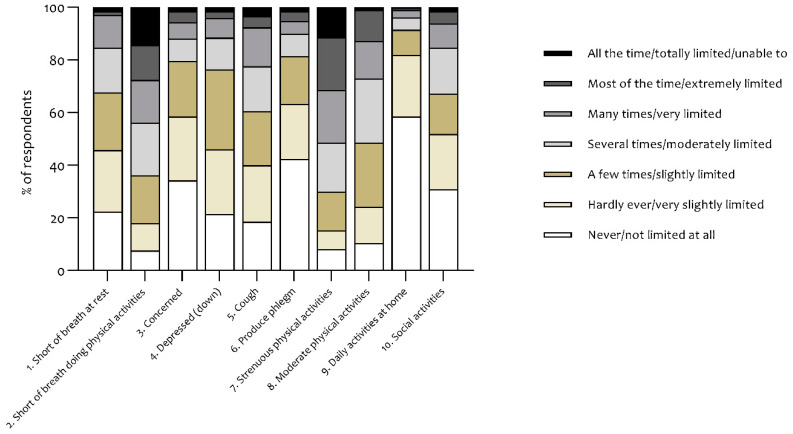
Distribution of responses to all of the ten CCQ items.

The mean CCQ score was 2.01 ± 0.98 points, while 123 respondents (59%) had a total score ≥1.9 points. There were no statistically significant differences between the diagnosis groups (Table 2). The symptoms and functional state domains were equally affected, demonstrating mean scores of 2.13 ± 1.12 and 2.12 ± 1.22 points, respectively. CCQ items 2 and 7 had the greatest impact on total scores, with 64% of the patients having shortness of breath during physical activities at least on a regular basis (i.e., several times) and 70% of the patients being at least moderately limited due to their respiratory symptoms during strenuous physical activities, respectively (Figure 2).

### 3.3. Associations between Generic and Respiratory-Specific Quality of Life

**Figure 3:**
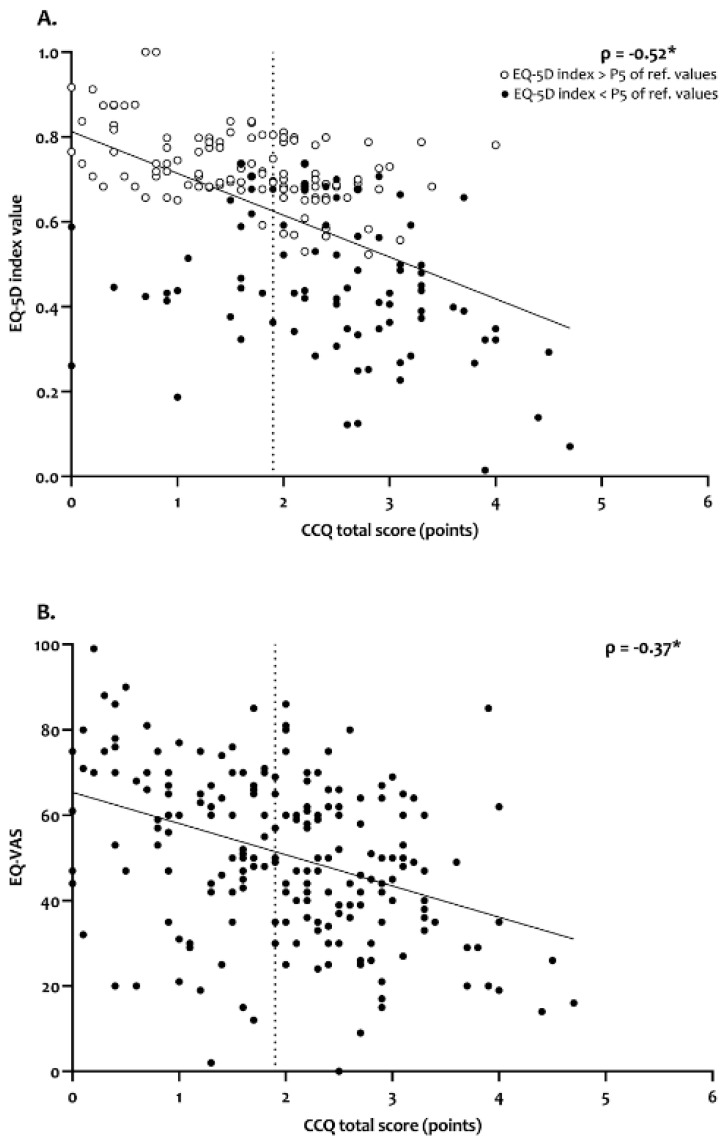
Association between EQ-5D index scores (**A**) and EQ-VAS scores (**B**) with CCQ total scores. Spearman’s rank correlation ρ is presented for both associations. On the horizontal axis, the CCQ total score cut off of 1.9 points is displayed [15]. * = *p* < 0.001. Abbreviations: EQ-5D-5L = five-level EQ-5D; VAS = visual analogue scale; P5 = fifth percentile; CCQ = Clinical COPD Questionnaire.

The correlation between EQ-5D index score/EQ-VAS score and CCQ total score was moderate (r = −0.524 and r = −0.374; both *p* < 0.001; Figure 3). The complete correlation matrix between the EQ-5D-5L dimensions and CCQ domain scores is shown in Table S2. Furthermore, 52% of the patients with a CCQ total score ≥1.9 points [15] had an EQ-5D index value below the 5th percentile of the normative values [13].

## 4. Discussion

Our study is the first to report that both generic and respiratory-specific quality of life is affected in non-hospitalized patients with COVID-19, approximately three months after the first symptoms related to COVID-19 appeared.

In the current study, the mean EQ-5D index value and EQ-VAS were 0.62 and 51, respectively, whereas the mean EQ-5D index value and EQ-VAS score for a representative sample of the general population have been estimated at 0.88 and 72, respectively [13]. When compared to EQ-5D values in patients with respiratory diseases such as COPD (mean EQ-5D index score: 0.51–0.68 and mean EQ-VAS: 61–63) [17,18] and asthma (mean EQ-5D index: 0.77–0.88 and mean EQ-VAS: 57–67) [19,20], the current findings demonstrate that generic quality of life is equally affected in non-hospitalized COVID-19 patients with persistent complaints three months after COVID-19 symptom onset. The same is true for respiratory-specific quality of life. The mean CCQ total score in the present study was 2.0 points, which is highly comparable to the CCQ score of 2.1 reported in a study including on >2000 patients with COPD [15]. The fact that our sample of non-hospitalized patients with COVID-19, with a mean age of only 45 years and of which the majority had no pre-existing comorbidities, demonstrates a respiratory-specific quality of life that is equivalent to (or even worse than) patients with advanced chronic lung disease, emphasizes the substantial persistent burden of COVID-19 in this widely overlooked population.

Little is known about long-term recovery from COVID-19 disease and studies about quality of life in these patients are lacking. A study by Wong and colleagues (2020) showed that over half of previously hospitalized COVID-19 survivors had a lower quality of life (measured with EQ-5D) three months after symptom onset compared to age-matched norms of the Canadian population [7]. Our non-hospitalized population showed similar results, with 40% of the patients reporting a generic quality of life below the fifth percentile of reference values. Looking at influenza A (H7N9) survivors, quality of life scores, as assessed with the Short-Form 36 questionnaire, were also lower at 3 months post-hospitalization compared with healthy controls and did not even improve during 24 months follow up [21].

Previous studies have found moderate to strong associations between disease-specific and generic health status questionnaires in respiratory patient populations such as asthma and COPD [18,20]. In the current population, a moderate correlation (−0.524) was found between EQ-5D index value and CCQ total score, which is in line with a previous study mapping the two instruments and reporting a correlation of −0.514 [22] in patients with COPD. Nevertheless, the fact that these two types of instruments measure non-overlapping aspects of quality of life should be always kept in mind. Generic questionnaires cover several aspects of daily life and facilitate comparisons among different populations and/or diseases, which might be superior to adequately distinguish the current overlooked group of post-COVID-19 patients from the general population. However, the EQ-5D lacks symptom-specific measures and the fact that symptoms are majorly affecting health status in this population underlines the assumption that generic quality of life instruments may not be sensitive enough without a supplementary respiratory-specific, symptom-based quality of life assessment. On the other hand, it was demonstrated recently that the post-COVID-19 symptom burden shows large heterogeneity, including fatigue, sleeping problems, muscle weakness and pain [3]. Therefore, an instrument such as the CCQ, which largely focuses on (the consequences of) respiratory symptoms, might be insufficient to comprehensively assess quality of life in post-COVID-19 patients. Indeed, the current study showed that after three months, almost 60% of the non-hospitalized patients still had an impaired respiratory-specific quality of life. However, with an average number of six persistent symptoms three months after the first symptoms appeared (Table 1), we have to extend our view beyond just respiratory symptoms.

A limitation of the current study is the limited external validity due the fact that our sample was limited to one geographic location, consisted of mostly women with a non-test-confirmed diagnosis and only included patients who gathered in an online social support group specifically focused on persistent COVID-19-related symptoms. Although this probably overrepresents the female sex and overestimates the actual impact on quality of life in the general non-hospitalized COVID-19 population, it may also be a reliable representation of the current population of non-hospitalized COVID-19 survivors with persistent symptoms who have not been formally tested due to a limited testing capacity [23]. Obviously, much remains unknown about COVID-19, including differences in the impact on men and women. However, according to the weekly surveillance reports of the World Health Organization (WHO) [24] the number of hospitalizations, ICU admissions and deaths is higher in men, despite the fact that most of the reported cases are female. Our study also included patients with symptom-based or suspected COVID-19. As the COVID-19 testing capacity was too limited, not all patients were tested in the beginning of the pandemic. Furthermore, Greenhalgh et al., (2020) stated that false negative tests are common and it was suggested that a positive test for COVID-19 was not a prerequisite for diagnosis [23]. As demonstrated in the results, there were no statistically significant differences between the diagnosis groups regarding clinical characteristics, number of symptoms and EQ-5D and CCQ outcomes, which indicates that even non-tested patients with suspected COVID-19 may still experience serious symptoms and an impaired quality of life months after the infection and require further attention. This study underlines the need for future studies to gain a comprehensive picture of the impact COVID-19 has had on longer-term health outcomes.

Furthermore, there is no information regarding pulmonary function in our patients, which limits the discriminative ability between patients with an impaired or preserved pulmonary function and its association with respiratory-specific quality of life. However, it has been demonstrated that pulmonary function is generally impaired in patients hospitalized with COVID-19 [8,9]. In the current study, the CCQ, which is generally used in respiratory disease patients, was used to assess respiratory-specific quality of life in patients with COVID-19. Obviously, although many different assessment tools (e.g., COPD Assessment Test, St George Respiratory Questionnaire) have similar psychometric properties, the CCQ was chosen mainly due to the fact it is a short, reliable, valid and easy-to-administer questionnaire and is preferred by most patients [25].

In conclusion, this is the first comparison of respiratory-specific and generic quality of life in non-hospitalized patients with confirmed/suspected COVID-19 using two validated measures. Hence, we can derive important insights on how different instruments affect the evaluation of quality of life in this population. Given the moderate association and limited discriminative ability of both questionnaires, the combined usage of the EQ-5D and the CCQ can be regarded as a promising approach to best describe quality of life in patients with COVID-19. Future research should focus on the possibilities and attributes of a combined instrument to depict quality of life in patients recovering from COVID-19, who have been symptomatic for an extended period of time. This might support further recommendations on how impaired quality of life in pandemic viral infections should be addressed to adequately assess disease burden. Indeed, our data underline the impact of COVID-19 and highlights the importance of a comprehensive assessment of possible underlying causes. This will help to reveal patients’ needs in order identify relevant rehabilitative interventions to effectively restore health and quality of life.

## Figures and Tables

**Table 1:** General characteristics.

	All Subjects (*n* = 210)	Confirmed COVID-19 (*n* = 49)	Symptom-Based COVID-19 (*n* = 105)	Suspected COVID-19 (*n* = 56)
Female, *n* (%)	184 (87.6)	45 (91.8)	93 (88.6)	46 (82.1)
Age (years)	45 ± 11	43 ± 10	45 ± 10	46 ± 12
18–39 years, *n* (%)	71 (33.8)	22 (44.9)	34 (32.4)	15 (26.8)
40–59 years, *n* (%)	116 (55.2)	24 (49.0)	60 (57.1)	32 (57.1)
60 + years, *n* (%)	23 (11.0)	3 (6.1)	11 (10.5)	9 (16.1)
Body Mass Index (kg/m^2^)	25.6 ± 4.3	26.3 ± 3.8	25.5 ± 4.5	25.0 ± 4.1
Pre-existing comorbidities				
None, *n* (%)	129 (61.4)	33 (67.3)	60 (57.1)	36 (64.3)
One, n (%)	49 (23.3)	6 (12.2)	31 (29.5)	12 (21.4)
Two or more, *n* (%)	32 (15.2)	10 (20.4)	14 (13.4)	8 (14.3)
Symptoms				
Time since first symptoms (days)	79 ± 17	74 ± 19	78 ± 15	84 ± 19 *
Symptoms during the infection, *n* (%)	14 (11–17)	14 (11–17)	14 (11–18)	14 (9–16)
Symptoms at this moment (after mean of 79 days), *n* (%)	6 (4–9)	6 (4–8)	7 (5–10)	6 (4–9)
Marital Status				
Alone, *n* (%)	43 (20.5)	11 (22.4)	19 (18.1)	13 (23.2)
Married/Living together, *n* (%)	147 (70.0)	34 (69.4)	77 (73.3)	36 (64.3)
Divorced, *n* (%)	18 (8.6)	4 (8.2)	8 (7.6)	6 (10.7)
Widow(er), *n* (%)	2 (1.0)	0 (o)	1 (1.0)	1 (1.8)
Level of Education				
Low, *n* (%)	15 (7.1)	1 (2.0)	6 (5.7)	8 (14.3)
Medium, *n* (%)	61 (29.0)	14 (28.6)	33 (31.4)	14 (25.0)
High, *n* (%)	133 (63.3)	33 (67.3)	66 (62.9)	34 (60.7)
Not specified, *n* (%)	1 (0.5)	1 (2.0)	0 (0)	0 (0)
Smoking Status				
Current smoker, *n* (%)	16 (7.6)	4 (8.2)	10 (9.5)	7 (12.5)
Stopped smoking after COVID-19, *n* (%)	39 (18.6)	14 (28.6)	31 (29.5)	11 (19.6)
Never smoker, *n* (%)	155 (73.8)	31 (63.3)	64 (61.0)	38 (67.9)

Variables are presented as proportions, the mean ± standard deviation or the median (interquartile range), as appropriate. Abbreviations: COVID-19 = coronavirus disease 2019, *n* = number, and kg/m^2^ = kilogram body weight divided by squared height in meters. * = *p* ≤ 0.05 (between group difference).

**Table 2:** EQ-5D-5L and CCQ outcomes.

Variables	All Subjects (*n* = 210)	Confirmed COVID-19 (*n* = 49)	Symptom-Based COVID-19 (*n* = 105)	Suspected COVID-19 (*n* = 56)
Five-level EQ-5D (EQ-5D-5L)				
EQ-5D index-score (range: −0.329–1)	0.62 ± 0.19	0.63 ± 0.20	0.61 ± 0.17	0.61 ± 0.20
Index-score <P5 of reference values, *n* (%) [13]	84 (40.0)	17 (34.7)	44 (41.9)	23 (41.1)
EQ-5D dimension scores (range: 1–5 *)				
Mobility	2.42 ± 1.04	2.39 ± 1.02	2.56 ± 1.02	2.20 ± 1.07
Self-care	1.19 ± 0.54	1.16 ± 0.51	1.24 ± 0.61	1.13 ± 0.38
Usual activities	2.95 ± 0.98	2.96 ± 1.00	3.09 ± 0.92	2.70 ± 1.04
Pain/Discomfort	2.87 ± 0.77	2.84 ± 0.80	2.90 ± 0.75	2.84 ± 0.80
Anxiety/Depression	2.10 ± 0.96	1.96 ± 0.87	2.05 ± 0.96	2.30 ± 1.03
EQ-VAS score (range: 0–100)	50.71 ± 18.87	51.27 ± 17.86	50.26 ± 20.06	51.09 ± 17.71
Clinical COPD Questionnaire (CCQ)				
CCQ total score (range: 0–6)	2.01 ± 0.98	2.00 ± 0.96	2.05 ± 0.95	1.93 ± 1.05
CCQ total score ≥1.9, *n* (%) [15]	123 (58.6)	29 (59.2)	67 (63.8)	27 (48.2)
CCQ domain scores (range: 0–6)				
Symptoms (items 1, 2, 5, 6)	2.13 ± 1.12	2.14 ± 1.09	2.18 ± 1.08	2.02 ± 1.23
Functional state (items 7, 8, 9, 10)	2.12 ± 1.22	2.10 ± 1.34	2.23 ± 1.20	1.90 ± 1.14
Mental state (items 3, 4)	1.56 ± 1.31	1.50 ± 1.13	1.45 ± 1.30	1.81 ± 1.47
CCQ item scores (range: 0–6)				
CCQ-1 Shortness of breath at rest	1.84 ± 1.46	1.84 ± 1.49	1.81 ± 1.43	1.89 ± 1.52
CCQ-2 Shortness of breath doing physical activities	3.24 ± 1.80	3.37 ± 1.89	3.26 ± 1.77	3.09 ± 1.78
CCQ-3 Concerned about getting a cold or your breathing getting worse	1.47 ± 1.51	1.39 ± 1.26	1.31 ± 1.44	1.82 ± 1.79
CCQ-4 Depressed (down) because of your breathing problems	1.65 ± 1.40	1.61 ± 1.32	1.58 ± 1.40	1.80 ± 1.49
CCQ-5 Cough	2.14 ± 1.62	2.12 ± 1.58	2.23 ± 1.60	2.00 ± 1.72
CCQ-6 Produce phlegm	1.30 ± 1.51	1.22 ± 1.54	1.43 ± 1.55	1.11 ± 1.38
CCQ-7 Strenuous physical activities	3.41 ± 1.74	3.57 ± 1.63	3.53 ± 1.86	3.04 ± 1.58
CCQ-8 Moderate physical activities	2.58 ± 1.51	2.63 ± 1.54	2.74 ± 1.49	2.21 ± 1.47
CCQ-9 Daily activities at home	0.73 ± 1.10	0.65 ± 1.27	0.74 ± 1.02	0.77 ± 1.11
CCQ-10 Social activities	1.73 ± 1.61	1.55 ± 1.76	1.89 ± 1.61	1.59 ± 1.46

Variables are presented as proportions or the mean ± standard deviation, including cut-off values for both questionnaires. Higher EQ-5D index and EQ-VAS scores indicate better generic quality of life, whereas higher CCQ scores indicate worse respiratory-specific quality of life. Abbreviations: *n* = number; VAS = visual analogue scale; P5 = fifth percentile. * The five EQ-5D dimensions have five levels of response (1: no problems, 2: slight problems, 3: moderate problems, 4: severe problems, and 5: extreme problems/unable to).

## Supplementary Materials

The following are available online at https://www.mdpi.com/2077-0383/9/12/3993/s1, Table S1: EQ-5D-5L frequencies and proportions reported by dimension and level. Table S2: Correlation matrix between the CCQ and the EQ-5D.
