# Supplementary material for: Generic and Respiratory-Specific Quality of Life in Non-Hospitalized Patients with COVID-19
Source: J Clin Med. 2020 Dec 9;9(12):3993. doi: 10.3390/jcm9123993 (PMC7764406; doi:10.3390/jcm9123993)
Supplement: Supplementary file 1 [file jcm-09-03993-s001.pdf]

Supplementary file.

Table S1. EQ-5D-5L frequencies and proportions reported by dimension and level.

	Mobility n (%)	Self-care n (%)	Usual activities n (%)	Pain/ Discomfort n (%)	Anxiety/ Depression n (%)
**Level 1** (No problems)	44 (21.0)	180 (85.7)	13 (6.2)	6 (2.9)	63 (30.0)
**Level 2** (Slight problems)	69 (32.9)	23 (11.0)	56 (26.7)	58 (27.6)	86 (41.0)
**Level 3** (Moderate problems)	68 (32.4)	5 (2.4)	80 (38.1)	106 (50.5)	42 (20.0)
**Level 4** (Severe problems)	22 (10.5)	1 (0.5)	50 (23.8)	38 (18.1)	16 (7.6)
**Level 5** (Extreme problems / unable to do)	7 (3.3)	1 (0.5)	11 (5.2)	2 (1.0)	3 (1.4)

Table S2. Correlation matrix between the CCQ and the EQ-5D.

Item	Description	EQ-5D index	Mobility	Self-care	Usual activities	Pain/ Discomfort	Depression/ Anxiety	EQ-VAS
**CCQ total**		-.524*	.377*	.277*	.384*	.468*	.308*	-.374*
**Symptoms**	CCQ-1. CCQ-2. CCQ-5. CCQ-6	-.301*	.271*	.202*	.200*	.322*	.114	-.227*
**Functional state**	CCQ-7. CCQ-8. CCQ-9. CCQ-10	-.543*	.496*	.342*	.526*	.476*	.197*	-.448*
**Mental state**	CCQ-3. CCQ-4	-.362*	.035	.053	.091	.297*	.525*	-.156*
**CCQ-1**	Short of breath at rest	-.263*	.152*	.134	.140*	.285*	.221*	-.252*
**CCQ-2**	Short of breath doing physical activities	-.330*	.344*	.185*	.213*	.370*	.155*	-.259*
**CCQ-3**	Concerned about getting a cold or your breathing getting worse	-.289*	-.002	.041	.056	.299*	.434*	-.122
**CCQ-4**	Depressed (down) because of your breathing problems	-.363*	.079	.065	.109	.237*	.509*	-.162*
**CCQ-5**	Cough	-.095	.128	.078	.089	.133	-.024	-.060
**CCQ-6**	Produce phlegm	-.132	.145*	.200*	.111	.100	-.035	-.104
**CCQ-7**	Strenuous physical activities	-.434*	.394*	.194*	.365*	.398*	.195*	-.314*
**CCQ-8**	Moderate psychical activities	-.484*	.545*	.303*	.545*	.434*	.149*	-.437*
**CCQ-9**	Daily activities at home	-.368*	.264*	.411*	.324*	.347*	.128	-.323*
**CCQ-10**	Social activities	-.478*	.382*	.268*	.464*	.368*	.189*	-.392*

* = p≤0.05. Abbreviations: VAS = Visual Analogue Scale; CCQ = Clinical COPD Questionnaire .
